# Tremors and Health-Related Quality of Life in Liver Transplant Recipients: Post-hoc Analysis of a Multicenter, Randomized, Controlled Trial Comparing a Life Cycle Pharma-Tacrolimus Regimen and Extended-Release Tacrolimus Regimen

**DOI:** 10.3389/ti.2025.14189

**Published:** 2025-04-28

**Authors:** M. B. Mulder, J. J. Busschbach, B. van Hoek, W. G. Polak, I. P. J. Alwayn, B. C. M. de Winter, S. Darwish Murad, E. Verhey-Hart, L. Elshove, N. S. Erler, D. A. Hesselink, C. M. den Hoed, H. J. Metselaar

**Affiliations:** ^1^ Department of Hospital Pharmacy, Erasmus MC, University Medical Center Rotterdam, Rotterdam, Netherlands; ^2^ Erasmus MC Transplant Institute, Rotterdam, Netherlands; ^3^ Section of Medical Psychology and Psychotherapy, Department of Psychiatry, Erasmus MC, University Medical Center Rotterdam, Rotterdam, Netherlands; ^4^ Department of Gastroenterology and Hepatology, LUMC, Leiden University Medical Center, Leiden, Netherlands; ^5^ Department of Surgery, Division of HPB and Transplant Surgery, Erasmus MC, University Medical Center Rotterdam, Rotterdam, Netherlands; ^6^ Department of Surgery, LUMC, Leiden University Medical Center, Leiden, Netherlands; ^7^ Department of Gastroenterology and Hepatology, Erasmus MC, University Medical Center Rotterdam, Rotterdam, Netherlands; ^8^ Department of Biostatistics, Erasmus MC, University Medical Center Rotterdam, Rotterdam, Netherlands; ^9^ Department of Epidemiology, Erasmus MC, University Medical Center Rotterdam, Rotterdam, Netherlands; ^10^ Department of Internal Medicine, Division of Nephrology and Transplantation, Erasmus MC, University Medical Center Rotterdam, Rotterdam, Netherlands

**Keywords:** liver transplantation, immunosuppressive therapy, tacrolimus, tremors, healthrelated quality of life

## Abstract

We investigated whether life cycle pharma (LCP)-tacrolimus compared to extended-release (ER)-tacrolimus results in a difference in severity of tremors and HRQoL. In this multi-center, open-label, randomized, controlled trial, 108 patients were randomized in a 1:1 ratio to either LCP-tacrolimus regimen or ER-tacrolimus regimen after transplantation. HRQoL was assessed with the EQ-5D-5L and SF-36 questionnaire (two generic HRQoL instruments) and the quality of life in essential tremor (QUEST) questionnaire (domain specific HRQoL instrument). The EQ-5D-5L scores were translated to the societal values. We examined the HRQoL over the course of the study by fitting generalized mixed effect models. In total, 105 patients were included, 53 to the LCP- and 52 to the ER-tacrolimus regimen. Baseline questionnaires were available for every LT recipient. At 12 months 25% [10/40], 95% confidence interval (CI) 14.2%–40.2% of the LT recipients in the LCP-tacrolimus regimen group experienced tremors compared to 30.4% [14/46], 95%-CI 19.1%–44.8% of the LT recipients in the ER-tacrolimus regimen group; risk difference: 0.054; 95%-CI −0.151–0.249; p = 0.63. No statistically significant differences in HRQoL were seen between the two regimens. We could not demonstrate differences in the HRQoL or occurrence of tremors between LCP-tacrolimus and ER-tacrolimus regimens

## Graphical Abstract

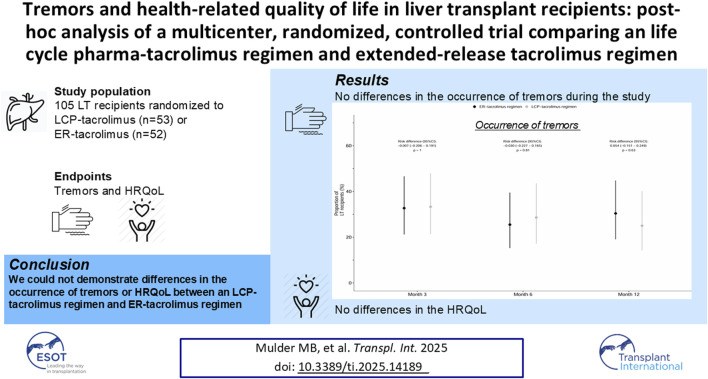

## Introduction

Liver transplantation (LT) is the preferred treatment for patients with end-stage liver disease and unresectable hepatocellular carcinoma (HCC). After LT, health-related quality of life (HRQoL) generally reaches a level like the general population, except for the aspect of physical functioning [1, 2]. In general, transplant recipients need to take lifelong immunosuppressive agents. These agents are not free from side effects with everyday challenges to the quality of life [3]. Therefore, the choice of immunosuppressive agents may impact the HRQoL of LT recipients.

Tacrolimus is the cornerstone of the immunosuppressive regimen after LT and belongs to the class of calcineurin inhibitors (CNIs) [4]. CNIs are associated with neurotoxicity and affect the central and peripheral nervous systems [5, 6]. Peripheral tremors are the most frequently occurring neurological side effect and affect 30%–55% of solid organ transplant recipients [7]. Tacrolimus exposure (whole blood trough concentrations) are associated with the severity of tremors [7].

Life cycle pharma (LCP)-tacrolimus, (Envarsus^®^; Chiesi Farmaceutici S.p.A.) is a prolonged-release tacrolimus formulation utilizing a new drug delivery technology (MeltDose) [8, 9]. This formulation has lower peak-through blood level fluctuations and a higher bioavailability compared to the other tacrolimus formulations, resulting in a lower dose requirement to reach the intended tacrolimus exposure [8, 10]. Therefore, it is hypothesized that LCP-tacrolimus could reduce the frequency and severity of peripheral tremors.

Several studies investigated the change in tremor severity after switching from tacrolimus twice-daily capsules (Prograf^®^, Astellas Pharma) or extended-release (ER)-tacrolimus (Advagraf^®^, Astellas Pharma) to LCP-tacrolimus once-daily tablets in kidney transplant recipients [11, 12]. These studies found that patients on LCP-tacrolimus experienced significant improvement of tremor and QoL post-switch to LCP-tacrolimus irrespective of the previous tacrolimus formulation administered. However, a major limitation of these non-randomized, uncontrolled post-switch studies is the fact that only kidney transplant patients were included who already experienced a clinically significant tremor observed by a healthcare provider or by patient complaint. Up until now no head-to-head comparison between the two once-daily tacrolimus formulations has been performed.

The aim of this post-hoc analysis of our multicenter, randomized, controlled trial (MOTTO study) was to investigate whether an LCP-tacrolimus regimen compared to an ER-tacrolimus regimen results in a difference in the severity of tremors and HRQoL.

## Materials and Methods

### Study Design and Participants

An extensive description of the MOTTO study design has been published previously [13]. In brief, from day 5 after LT patients received twice-daily, immediate-release (IR) tacrolimus. After achieving stable tacrolimus trough levels between 8–10 μg/L, patients were randomized in a 1:1 ratio to either a LCP-tacrolimus regimen or an ER-tacrolimus regimen. To prevent for to toxicity (renal insufficiency or tremors), rejection or to prevent recurrence of hepatocellular carcinoma: in the LCP-tacrolimus group 19 patients used a combination regimen of LCP-tacrolimus with mycophenolic acid and 2 LT recipients to LCP-tacrolimus and sirolimus. In the ER-tacrolimus group 22 LT recipients used to a regimen of ER-tacrolimus and mycophenolic acid. The study was performed at two centers in the Netherlands: The Erasmus MC, University Medical Center Rotterdam, Rotterdam, Netherlands and Leiden University Medical Center, Leiden, Netherlands. The study was approved by the institutional Ethical Committees of these institutions, registered in the EudraCT database (EudraCT: 2018-002856-34) and conducted in accordance with the latest version of the declaration of Helsinki. The inclusion period ran from April 2019 until October 2021.

### Patient-Reported Outcomes

The evaluation of the severity of tremors and the HRQoL comprised a pre-defined secondary objective of the MOTTO study. The MOTTO study was initially designed to investigate whether LCP-tacrolimus compared to ER-tacrolimus results in a difference in the prevalence of post-transplant diabetes mellitus, new onset hypertension and chronic kidney disease at 12 months after transplantation.

### HRQoL and Severity of Tremor Assessments

HRQoL was assessed with the validated Dutch version of the EQ-5D-5L questionnaire and the SF-36 questionnaire (two generic HRQoL instruments) and the quality of life in essential tremor (QUEST) questionnaire (a domain specific HRQoL instrument). The questionnaires were distributed at the day of randomization, month 3, 6, and 12.

The EQ-5D-5L questionnaire is based on a descriptive system that defines health in terms of 5 states: Mobility, Self-Care, Usual Activities, Pain/Discomfort, and Anxiety/Depression [14]. Each dimension has 5 response categories corresponding to no problems, slight problems, moderate problems, severe problems, and extreme problems. EQ-5D-5L scores were transformed to societal values based on the Dutch tariff for the EQ-5D-5L established by Versteegh *et al*. [15].

In the EQ-5D-5L questionnaire, the respondents’ overall health on the day of the interview (patient’s self-rated HRQoL scores) was rated on a 0–100 hash-marked, vertical visual analogue scale (EQ-VAS). The threshold for the minimally important difference (MID), indicating a clinical meaningful improvement, in the EQ-VAS score was defined as ≥7 points [16].

The SF-36 questionnaire contains 36 items grouped in eight domains: physical functioning, role limitation-physical, pain, general health, energy/fatigue, social functioning, emotional wellbeing, role limitation-emotional. Each domain is scored between 0 and 100 points, with higher scores indicating better HRQoL.

The QUEST questionnaire is a self-administered questionnaire with 30 items on a five-point scale (0–4), corresponding to the frequency (never, rarely, sometimes, frequently, always) with which tremor is perceived to currently impact five domains: physical, psychosocial, communication, hobbies/leisure and work/finance [17, 18]. The score on each domain is expressed as a percentage of the total score possible on that domain, with a higher score indicating greater dissatisfaction with that domain of QoL. A total score was computed by calculating the mean of the five domain scores.

Given that the QUEST is “domain specific” for “patients with essential tremors,” this questionnaire is most likely more sensitive than the generic EQ-5D-5L and SF-36. The value of those two questionnaires is the ability to formulate “values” of quality of life for cost effectiveness analysis, and these generic questionnaires can measure side effects outside the measuring domain of the QUEST.

### Management of Tremors

In this study treating physicians were allowed to apply the current common practice in order to manage the severity of tremors. This includes either reduce the dose of tacrolimus while maintaining the LT recipient on monotherapy tacrolimus or start combination therapy of tacrolimus and another immunosuppressive agent. No comedication to actively treat tremors such as beta-blockers or anticonvulsants were allowed to start for the treatment of tremors.

### Data Collection

Variables collected included recipient socio-demographic, clinical and transplantation parameters, the HRQoL and tremor severity and trough levels tacrolimus.

### Statistical Analysis

The HRQoL analysis included all patients within the MOTTO study who responded to at least one questionnaire. The EQ-5D-5L, SF-36 and QUEST questionnaire included in the analysis missed <5% based on the total number of measurements across all patients and questions. The missing data were considered as missing completely at random.

Two generalized linear mixed effect models were fitted to examine the HRQoL (EQ-VAS and the societal values of the EQ-5D-5L) over the course of the study. The models included covariates shown or suggested to be relevant: time since transplantation, study group, tacrolimus trough concentrations, kidney function, hemoglobin, recipient age and sex, primary disease, diabetes mellitus and hypertension pre-transplantation as well as the interaction between visit and the study group. Participant specific random intercepts were included to account for correlation among repeated measurement nested within each participant. Natural cubic splines were used to model the potentially nonlinear trajectories of the EQ-VAS and societal values of the EQ-5D-5L over time. The need for these splines was evaluated using likelihood-ratio tests. Splines provide a convenient non-parametric way to flexibly model (potentially) non-linear associations in regression models. Instead of using one polynomial (e.g., a quadratic or cubic function) that spreads over the whole range of the covariate, splines use a set of several polynomial functions that are defined over smaller intervals. This allows the resulting fit to be more flexible and less influenced by outliers than when using a single polynomial. To visualize the estimated associations, the expected HRQoL across the course of the study was calculated while fixing the values of all other covariates to the median or reference category.

Secondary endpoints were analyzed using the Pearson’s Chi-square test or Mann-Whitney U Test. Confidence intervals for binomial proportions were calculated using the binconf package for R software. For all statistical tests, a (two-sided) p-value of <0.05 was considered to indicate statistical significance.

All data were collected in CastorEDC and analysis was conducted with R software (version 4.2.1) [19, 20].

## Results

### Patient and Treatment Characteristics

**FIGURE 1:**
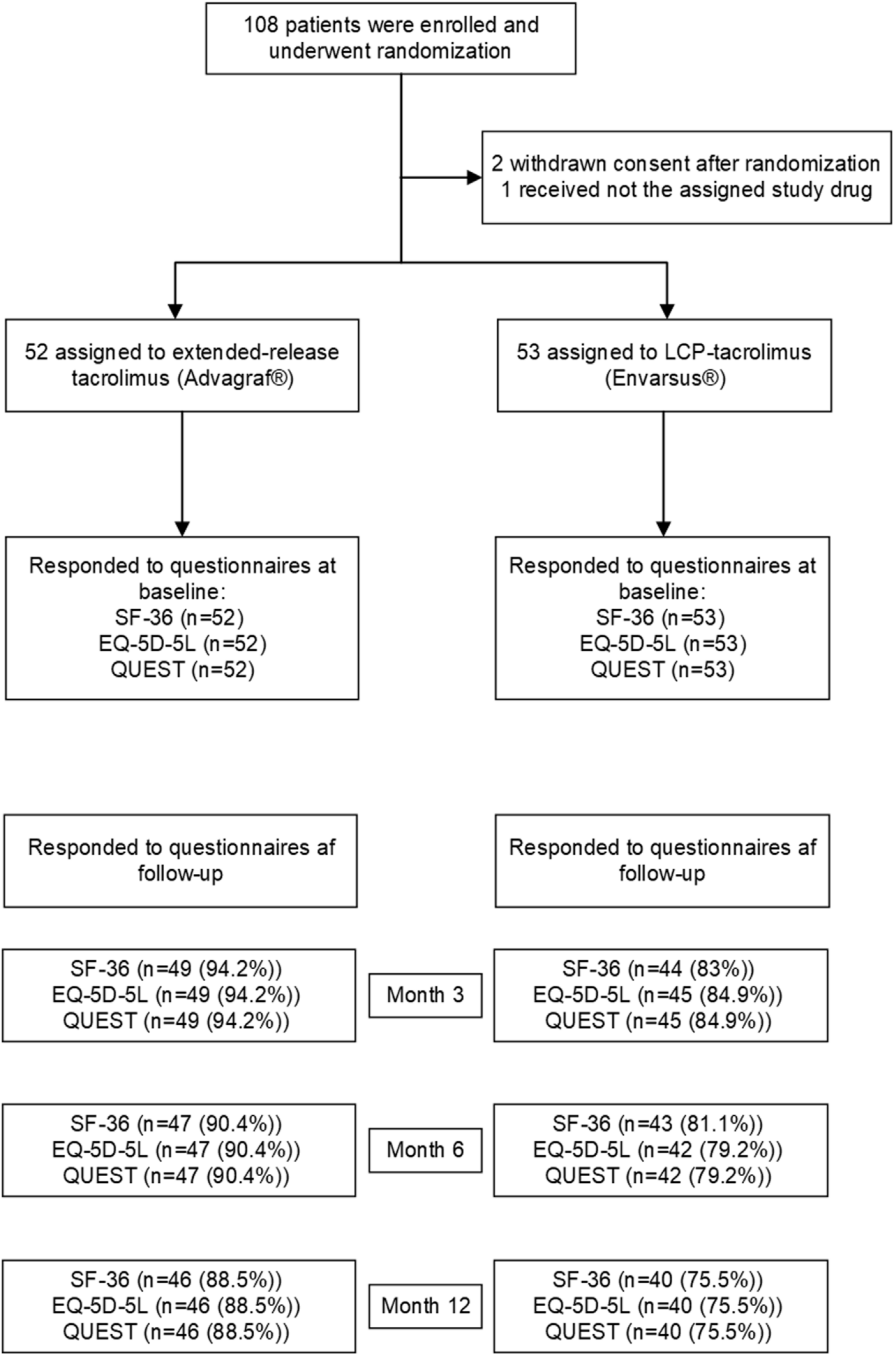
Enrollment, randomization, and follow-up.

A total of 108 LT recipients was included and randomized. No LT recipient included was diagnosed with a neurological movement disorder pre-transplantation. At baseline, 100% of the LT recipients responded to the EQ-5D-5L, SF-36 and QUEST questionnaires. The response rate decreased during follow up to a minimum of 75.5% at the end of the study (Figure 1).

Table 1 shows the baseline characteristics. No relevant differences in the baseline characteristics for the EQ-5D-5L questionnaire existed between the two regimens. However, more LT recipients randomized to the LCP-tacrolimus regimen experienced tremors compared to the LT recipients randomized to the ER-tacrolimus regimen [30.2% (16/53), 95%-confidence interval (CI) 19.9%–44.3% *versus 19*.2% (10/52), 95%-CI 10.8%–31.9%]. The mean tacrolimus trough level at the day of randomization to the LCP-tacrolimus regimen was 7.5 ± 3.3 μg/L and in the ER-tacrolimus regimen 6.9 ± 3.1 μg/L, p = 0.38. LT recipients in the LCP-tacrolimus regimen were converted to that formulation after 11 days (IQR: 9.25–15.25 days) and in the ER-tacrolimus regimen, LT recipients were converted to that formulation after 13.5 days (IQR: 9–15.75 days).

**TABLE 1:** Baseline characteristics.

	Extended-release tacrolimus (n = 52)	LCP-tacrolimus (n = 53)
Recipient demographics at randomization		
Age, year (median, IQR)	58.50 (46.75–65.25)	56.50 (46.25–63)
Gender, male (n, %)	41 (78.8%)	35 (66%)
Primary Disease (n, %)		
Hepatocellular carcinoma	19 (36.5%)	12 (22.6%)
(Non)alcoholic steatohepatitis	7 (13.5%)	10 (18.9%)
Primary sclerosing cholangitis	10 (19.2%)	8 (15.1%)
Acute liver failure	3 (5.8%)	3 (5.7%)
Cryptogenic cirrhosis	3 (5.8%)	3 (5.7%)
Metabolic diseases	-	4 (7.5%)
Viral Hepatitis	3 (5.8%)	3 (5.7%)
Other^a^	7 (13.5%)	11 (20.8%)
Lab		
Hemoglobin, mmol/L (mean ± SD)	6.25 ± 0.90	6.13 ± 0.84
eGFR, mL/min/1.73m^2^ (mean ± SD)	82.08 ± 17.83	79.44 ± 20.43
Tacrolimus trough blood level, µg/L (mean ± SD)	6.94 ± 3.05	7.46 ± 3.28
Smoking (n, %)	11 (21.2%)	8 (14.8%)
Recipient demographics pre-transplantation		
Pre-existing Diabetes, Yes (n, %)	11 (21.2%)	13 (24.5%)
Pre-existing Hypertension, Yes (n, %)	17 (32.7%)	11 (20.8%)
EQ-5D-5L questionnaire		
VAS (mean ± SD) [ref: 0–100]	65 ± 15	58 ± 17
Societal values of the EQ-5D-5L based on the Dutch tariff for the EQ-5D-5L (median, IQR) [ref: −0.466–1]	0.53 (0.35–0.62)	0.56 (0.37–0.67)
QUEST questionnaire		
LT recipients and tremors before the start of study drug, Yes (n, %)	10 (19.2%)	16 (30.2%)
Hours of tremors per day (median, IQR)	1.0 (1.0–3.5)	4.0 (1.0–7.0)
Total score QUEST (median, IQR)	1.15 (0.28–3.33)	12.29 (1.25–23.96)

Abbreviations: eGFR, estimated glomerular filtration rate based on the CKD-EPI, formula; INR, international normalized ratio; SD, standard deviation; IQR, interquartile range; QUEST, quality of life in essential tremor.

^a^ Other includes: primary biliary cirrhosis, secondary biliary cirrhosis, autoimmune cirrhosis, cholangiocarcinoma, Caroli disease, polycystic liver disease, neuroendocrine tumor liver metastases.

### Tremors

**FIGURE 2:**
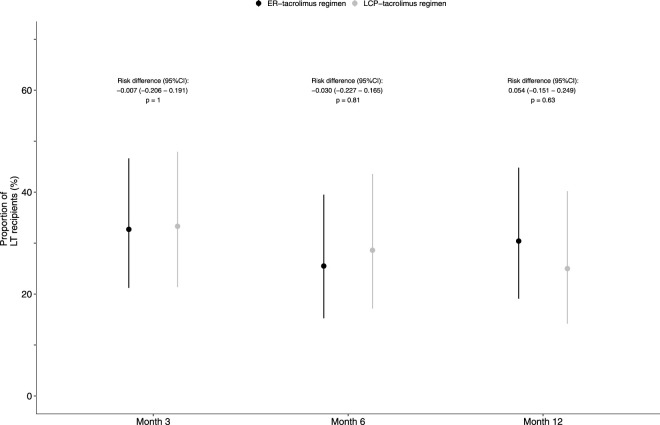
Proportion of LT recipients experiencing tremors during follow-up. The proportion of LT recipients with 95%-CI experiencing tremors during follow-up. At 12 months 25% [10/40], 95% confidence interval (CI) 14.2%–40.2% of the LT recipients in the LCP-tacrolimus group *versus* 30.4% [14/46], 95%CI 19.1%–44.8% of the LT recipients in the ER-tacrolimus group experienced tremors; risk difference: 0.054; 95%CI -0.151–0.249; p = 0.63.

Figure 2 shows the proportion of LT recipients experiencing tremors during study follow up. Supplementary Table S1 shows the QUEST questionnaire results and the tacrolimus levels of the LT recipients during the study. No statistically significant differences between the two regimens were found at 3, 6 and 12 months in the frequency and severity of tremors. At 12 months 25% [10/40], 95%-CI 14.2%–40.2% of the LT recipients in the LCP-tacrolimus regimen *versus* 30.4% [14/46], 95%-CI 19.1%–44.8% of the LT recipients in the ER-tacrolimus regimen experienced tremors; risk difference: 0.054; 95%CI -0.151–0.249; p = 0.63.

The mean tacrolimus trough level at 12 months in the LCP-tacrolimus regimen was statistically significantly higher compared to the ER-tacrolimus regimen: 7.6 ± 3.1 μg/L *versus* 6.3 ± 2.2 μg/L, p = 0.026. No statistically significant differences were observed in any of the five domains and the total score of the QUEST (Supplementary Figure S1). No relevant differences were found for tacrolimus levels versus regimen and the presence of tremors (Supplementary Figure S5).

Interestingly we did see effects of switching and dose reduction of tacrolimus. During the study period, some patients changed the immunosuppressive therapy because of tremors: one patient switched from ER-tacrolimus to LCP-tacrolimus, two patients switched from monotherapy LCP-tacrolimus to combination therapy of low-exposure LCP-tacrolimus with mycophenolic acid and one patient switched from monotherapy ER-tacrolimus to combination therapy of low-exposure ER-tacrolimus with mycophenolic acid. In all four LT recipients a reduction in the severity of tremors and an improved QUEST score after the switch was observed. The other patients experiencing tremors were managed by reducing the dose of the tacrolimus formulation while maintaining these patients on monotherapy with tacrolimus.

### Health-Related Quality of Life Outcomes

Supplementary Figure S2 shows the proportion of responses by level of severity for the EQ-5D-5L dimensions during the study period. Overall, patients reported the least issues in the states of Self-Care and Anxiety/Depression and the most problems in the states of Usual Activities and Pain/Discomfort. No evidence for differences between the study groups in any of the five domains was found.

**FIGURE 3:**
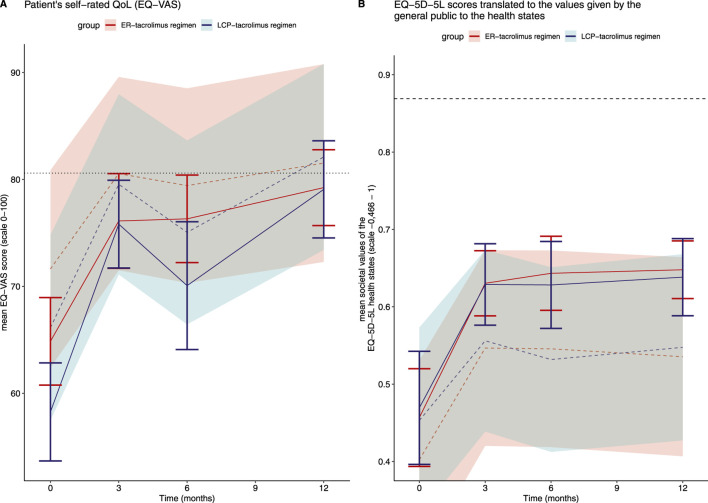
EQ-VAS score and EQ-5D-5L scores on the dimensions translated to the societal values. **(A)** Patient’s self-rated QoL (EQ-VAS) Group-wise mean EQ-VAS with 95%-confidence interval (CI) during the course of the study represented as solid lines. The dashed lines and shaded areas indicate the expected values and corresponding 95%-CI from the generalized mixed effect model (assuming the median or reference value for the continuous or categorical covariates, respectively: tacrolimus trough concentrations, kidney function, hemoglobin, recipient age and sex, primary disease, diabetes mellitus and hypertension pretransplantation as well as the interaction between visit and the study group). Dotted black line indicates the mean self-reported EQ-VAS score by the general Dutch population [15]. **(B)** EQ-5D-5L scores translated to the values given by the general public to the health states. Group-wise mean of the societal values of the EQ-5D-5L health states with 95%-confidence interval (CI) during the course of the study represented as solid lines. The dashed lines and shaded areas indicate the expected values and corresponding 95%-CI from the generalized mixed effect model (assuming the median or reference value for the continuous or categorical covariates, respectively tacrolimus trough concentrations, kidney function, hemoglobin, recipient age and sex, primary disease, diabetes mellitus and hypertension pretransplantation as well as the interaction between visit and the study group). Dotted black line indicates the mean EQ-5D-5L score given by the general Dutch population to the health states. [15]. Abbreviations: QoL, quality of life; VAS, visual analogue scale.

The likelihood-ratio tests indicated non-linear patient specific trajectories of HRQoL scores and the societal values of the EQ-5D-5L. No evidence was found for between-group differences over the course of the study based on the mixed effect models. The hemoglobin level was statistically significantly associated with a higher EQ-VAS and EQ-5D-5L score, whereas tacrolimus trough levels were statistically significantly associated with a lower EQ-VAS and EQ-5D-5L score (Supplementary Table S2). Figure 3 visualize the expected HRQoL scores and societal values of the EQ-5D-5L together with the corresponding observed values per time point and study group. At the end of the study, the patient’s self-rated HRQoL scores as expressed with the EQ-VAS approximate the mean self-reported EQ-VAS score by the general Dutch population. For both arms, the societal values of the EQ-5D-5L were below those of the general Dutch population.

LT recipients in both study groups achieved a clinically meaningful improvement (>7 points) in the EQ-VAS score at 12 months (LCP-tacrolimus: 20.8 points and ER-tacrolimus: 14.3 points difference with the moment of randomization).

Supplementary Figure S3 shows the results from the SF-36 questionnaire. Every domain of the SF-36 questionnaire improved during the follow-up. Most improvement was shown in the domains: physical functioning, social functioning and pain. No statistically significant differences were found between both study groups on any of the eight domains.

An analysis of the EQ-VAS score in relation to tremors did not show statistically significant differences between LT recipients with and without tremor as indicated by the QUEST questionnaire (Supplementary Figure S4).

## Discussion

This is the first head-to-head comparison of two once-daily tacrolimus formulation regimens (i.e., LCP- and ER-tacrolimus) evaluating tremor and HRQoL in the first year after liver transplantation. In this randomized controlled study, we found no significant differences in terms of both the frequency and severity of tremors and HRQoL in LT recipients using an LCP-tacrolimus regimen compared to an ER-tacrolimus regimen. HRQoL improved over the first 12 months after liver transplantation equally in both regimens.

The findings of our study on HRQoL are in line with several other studies showing that the HRQoL of LT recipients rapidly improves after LT [1, 21]. However, conflicting results regarding the use of different immunosuppressive agents and their impact on the HRQoL of LT recipients are reported [2, 22]. We did not find evidence for differences in the HRQoL between both once-daily formulations of tacrolimus, despite a different pharmacokinetic profile and assumed lower peak levels [8]. In addition, in a previous study by our research group we also did not find a difference in the HRQoL between two regimens with different immunosuppressive agents, namely, normal dose tacrolimus *versus* a combination of low dose tacrolimus and sirolimus [2]. During the current study follow-up, the EQ-VAS approximated the mean self-reported EQ-VAS score by the general Dutch population, whereas the societal values of the EQ-5D-5L were below those of the general Dutch population. Based on the limited available evidence, it remains to be determined whether different immunosuppressive agents and different formulations of immunosuppressive agents have a clinically relevant impact on the HRQoL of LT recipients.

We did not find a difference in frequency and severity of tremors between both once daily tacrolimus regimens. This in contrast with two clinical studies in kidney transplant recipients with pre-existing tremor showing less tremors after switching to LCP-tacrolimus [11, 23]. However, these two studies evaluated the conversion from different formulations of tacrolimus, i.e., twice daily immediate-released tacrolimus and extended-released tacrolimus to once-daily LCP-tacrolimus. To emphasize, we performed a head-to-head comparison between LCP- and ER-tacrolimus and not a conversion study, which could explain our different findings.

Based on our results, we cannot conclude whether LCP-tacrolimus or ER-tacrolimus is the best treatment option to reduce tremors. In daily clinical practice, when using tacrolimus, up to 50% of the solid organ transplant recipients experience tremors [6, 11]. In this study up to 34% of the LT recipients experienced tremors while using tacrolimus. A recent study showed that high tacrolimus trough concentrations were the main determinant of tremor [7]. Interestingly, in our study, the mean tacrolimus trough levels in the LCP-tacrolimus group were statistically significantly higher at the end of the study follow-up, while no differences in frequency and severity of tremor were found. This finding suggests that higher trough levels and a more stable pharmacokinetic profile of LCP-tacrolimus seems not to be related to the occurrence of tremors. Hypothetically more equal tacrolimus trough levels in both study groups might have resulted in less tremors in the LCP-tacrolimus group. Furthermore, previously we showed that the use of LCP-tacrolimus was associated with significantly lower rates of kidney dysfunction and hypertension [13].

Multiple factors have an influence on the appearance and severity of tremors such as the height of the tacrolimus trough levels, smoking, medical conditions (e.g., hypothyroidism and hypoglycemia) or the use of certain medications (e.g., beta-blockers, bronchodilators, anticonvulsants, antidepressants) [7, 24]. The number of LT recipients smoking was equally divided over both study groups. Unfortunately, adequate information regarding the use of concomitant drugs influencing tremors was not available. Since beta-blockers are occasionally prescribed to treat post-transplant hypertension the frequency and severity of tremors in this study might be underestimated.

Another study showed that severe tremor in solid organ transplant recipients was strongly and independently associated with lower physical and mental HRQoL [7]. We could not find lower HRQoL scores for LT recipients experiencing tremors compared to LT recipients without tremors.

A major strength of this study is the fact that this is a randomized controlled trial and not a conversion or switch study with a high response rate and longitudinal assessment of the HRQoL and severity of tremors. A limitation is the lack of statistical power in this post-hoc analysis. At 12 months, we found no statistical significant differences between the two treatment groups. However, we did find less tremors in the LCP-tacrolimus group. Potentially with more power and, as described above, more equal tacrolimus trough levels in both study groups we might have found a statistical significant and clinical relevant difference in the frequency of tremors in LT recipients on tacrolimus. An other limitation is the fact that the tremors reported by LT recipients were not evaluated by a physician using the Fahn-Tolosa-Marin tremor reporting scale. This tremor reporting scale was developed to quantify essential tremor severity and has been used in large trials for essential tremor. The QUEST questionnaire is a self-assessment and therefore the results regarding the severity of the tremor are not objectified.

We believe that reducing the dose of tacrolimus with or without adding another immunosuppressive agent is the way to go to reduce neurotoxicity in LT recipients.

In conclusion, based on this clinical study, an once-daily LCP-tacrolimus regimen is not associated with an improvement in the HRQoL or a reduction in the occurrence of tremors compared to an ER-tacrolimus regimen. Aiming for lower tacrolimus trough levels or exposure seems a better strategy to reduce the severity and frequency of tremors.

## Data Availability

The raw data supporting the conclusions of this article will be made available by the authors, without undue reservation.

## Abbreviations

CNI, Calcineurin inhibitors; eGFR, Estimated glomerular filtration rate; ER, Extended-release; HCC, Hepatocellular carcinoma; HRQoL, Health-related quality of life; IQR, Interquartile range; LCP, Life cycle pharma; LT, Liver transplantation; SAE, Serious adverse event; SD, Standard deviation; QUEST, Quality of life in essential tremor; VAS, Visual analogue scale.
