# Supplementary material for: Tremors and Health-Related Quality of Life in Liver Transplant Recipients: Post-hoc Analysis of a Multicenter, Randomized, Controlled Trial Comparing a Life Cycle Pharma-Tacrolimus Regimen and Extended-Release Tacrolimus Regimen
Source: Transpl Int. 2025 Apr 28;38:14189. doi: 10.3389/ti.2025.14189 (PMC12066294; doi:10.3389/ti.2025.14189)

# Capsule Sentence Summary

This is randomized controlled trial of a life cycle pharma (LCP) – tacrolimus regimen compared to a extended-release (ER) tacrolimus regimen, which showed no significant difference in the health-related quality of life or occurrence of tremors.

# Supplementary table 1. QUEST questionnaire results

|  | Month 3 | | Month 6 | | Month 12 | |
| --- | --- | --- | --- | --- | --- | --- |
|  | ER-tacrolimus regimen (n=49) | LCP-tacrolimus regimen (n=45) | ER-tacrolimus regimen (n=47) | LCP-tacrolimus regimen (n=42) | ER-tacrolimus regimen (n=46) | LCP-tacrolimus regimen (n=40) |
| Experienced tremors, Yes (n, %) | 16 (32.7) | 15 (33.3) | 12 (25.5) | 12 (28.6) | 14 (30.4) | 10 (25) |
| Severity tremors, hours/day (median, IQR) | 2.5 (1 – 4) | 2 (1 – 8.5) | 5 (1 – 9) | 1.5 (1 – 2.25) | 2 (1 – 8) | 1 (1 – 2) |
| Total QUEST score (median, IQR) | 5.56 (3.19 – 13.44) | 7.78 (2.22 – 17.5) | 5.78 (1.67 – 15.83) | 7.5 (1.87 – 16.56) | 6.32 (1.67 – 10) | 6.94 (1.46 – 18.75) |
| Tacrolimus trough level, µg/L (mean (SD)) | 6.59 (2.59) | 7.15 (1.99) | 6.8 (2.46) | 7.03 (2.23) | 6.27 (2.15) | 7.63 (3.14) |
| Number of recipients in target range tacrolimus, i.e. month 0-3 between 8-10 µg/L and after month 3 between 6-8 µg/L (n, %) | 7 (14.3) | 12 (26.7) | 20 (42.6) | 14 (33.3) | 17 (36.9) | 14 (35) |
| Number of recipients above target range tacrolimus, i.e. month 0-3 > 10 µg/L and after month 3 > 8 µg/L (n, %) | 4 (8.3) | 4 (9.8) | 12 (26.1) | 15 (36.6) | 7 (16.7) | 14 (36.8) |

Abbreviations: ER-tacrolimus, extended-release tacrolimus; SD, standard deviation, QUEST, Quality of Life in Essential Tremor

# Supplementary table 2. Results of the generalized mixed effect models for EQ-VAS and EQ-5D-5L scores on the dimensions translated to the societal values

|  | Model for EQ-VAS  (n=354) | | Model for EQ-5D-5L  (n=354) | |
| --- | --- | --- | --- | --- |
| **Fixed effects** | **Estimate** | **95%-CI** | **Estimate** | **95%-CI** |
| Intercept | 57.0 | 36.6 – 76.7 | 0.18 | -0.089 – 0.45 |
| ns (Visit, df=3)1 | 1.44 | -6.42 – 9.31 | 0.061 | -0.034 – 0.16 |
| ns (Visit, df=3)2 | 18.0 | 8.90 – 27.2 | 0.28 | 0.17 – 0.39 |
| ns (Visit, df=3)3 | 3.93 | -0.59 – 8.51 | 0.039 | -0.015 – 0.095 |
| Study group | -5.49 | -11.3 – 0.18 | 0.051 | -0.026 – 0.13 |
| Tacrolimus trough level | -0.78* | -1.29 – -0.25 | -0.0067* | -0.013 – -0.00043 |
| Kidney function | 0.05 | -0.06 – 0.16 | 0.00026 | -0.0012 – 0.0016 |
| Hemoglobin level | 2.40* | 0.81 – 4.06 | 0.021* | 0.00041 – 0.041 |
| Recipient age | -0.02 | -0.22 – 0.19 | 0.0019 | -0.00095 – 0.0048 |
| Recipient sex, male | -1.01 | -6.25 – 4.18 | 0.033 | -0.041 – 0.11 |
| Primary disease AIH | -2.52 | -13.8 – 8.86 | 0.088 | -0.073 – 0.25 |
| Primary disease ALF | -3.46 | -14.5 – 7.89 | 0.064 | -0.091 – 0.22 |
| Primary disease Cholestatic liver disease^†^ | -7.16 | -14.8 – 0.53 | 0.099 | -0.0083 – 0.21 |
| Primary disease Crytogenic cirrhosis | -8.83 | -19.3 – 1.61 | -0.024 | -0.17 – 0.12 |
| Primary disease HCC | -2.93 | -9.55 – 3.84 | 0.049 | -0.044 – 0.14 |
| Primary disease Metabolic disease | -12.8 | -25.9 – 0.27 | 0.043 | -0.14 – 0.23 |
| Primary disease other^‡^ | 5.19 | -4.87 – 15.2 | 0.061 | -0.080 – 0.20 |
| Primary disease Viral hepatitis | -4.19 | -14.3 – 5.99 | 0.097 | -0.047 – 0.24 |
| Pre-transplant Diabetes Mellitus | 2.59 | -3.16 – 8.44 | -0.013 | -0.094 – 0.068 |
| Pre-transplant Hypertension | -2.77 | -7.92 – 2.30 | -0.019 | -0.092 – 0.052 |
| Interaction between Visit and study group (df =3)1 | -4.89 | -16.2 – 6.5 | -0.064 | -0.20 – 0.075 |
| Interaction between Visit and study group (df =3)2 | 8.42 | -2.24 – 19.2 | -0.093 | -0.22 – 0.039 |
| Interaction between Visit and study group (df =3)3 | 2.58 | -3.76 – 8.97 | -0.016 | -0.092 – 0.062 |
| **Random effects** | **Variance** | **SD** | **Variance** | **SD** |
| Subject intercept | 97.07 | 9.85 | 0.021 | 0.15 |
| Residual | 121.9 | 11.0 | 0.017 | 0.13 |

Abbreviations: AIH, autoimmune hepatitis; ALF, acute liver failure; CI, confidence interval; HCC, Hepatocellular carcinoma; TAC, tacrolimus; SD, standard deviation; VAS, visual analogue scale; *indicates statistical significance

^†^Cholestatic liver disease includes: Primary biliary cholangitis, Primary sclerosing cholangitis, Caroli disease; biliary cirrhosis

*^‡^*Other includes: Cholangiocarcinoma; Neuroendocrine tumour; Polycystic liver disease

Two generalized mixed effect models were fitted, investigating the association between the EQ-VAS and the societal values of the EQ-5D-5L health states during the course of the study (values for the covariates: tacrolimus trough levels, kidney function, hemoglobin level, recipient age and sex, primary disease, pretransplant diabetes mellitus, pretransplant hypertension as well as the interaction between visit and the study group). Random participant effects were included to account for repeated measurement nested within each participant. The shape of the association with the EQ-VAS was investigated using natural cubic splines. The coefficients of the spline do not have a direct interpretation (see figures for interpretation). Missing data were considered as missing completely at random.

# Supplementary figure 1. QUEST questionnaire outcomes for every domain
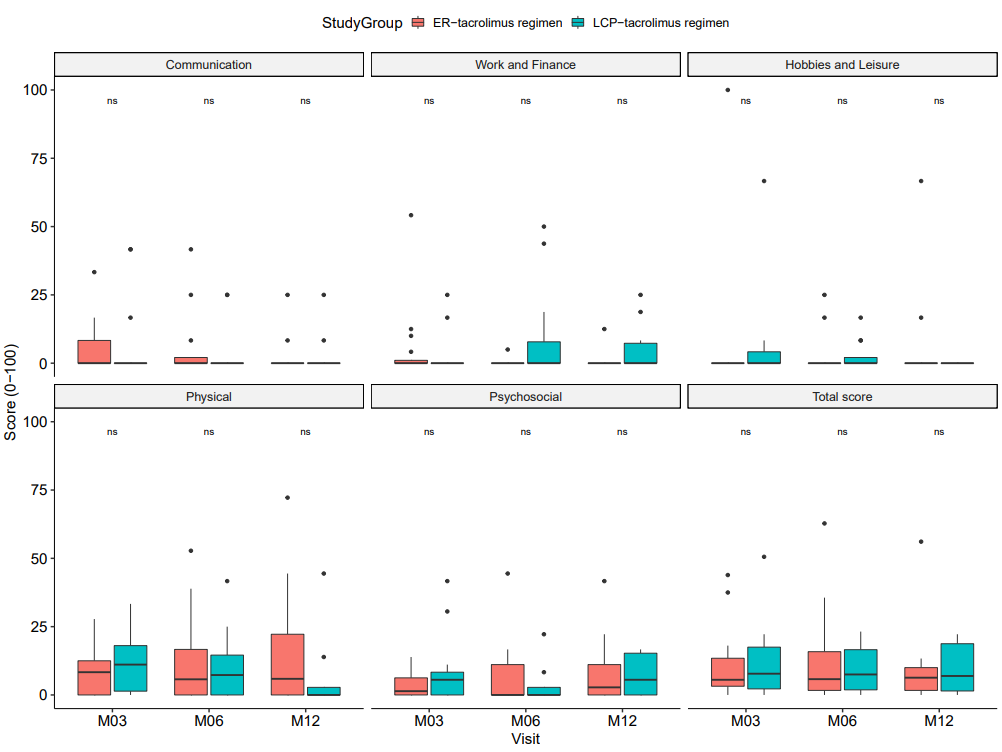


# Supplementary figure 2. The proportion of responses by level of severity for EQ-5D-5L dimensions


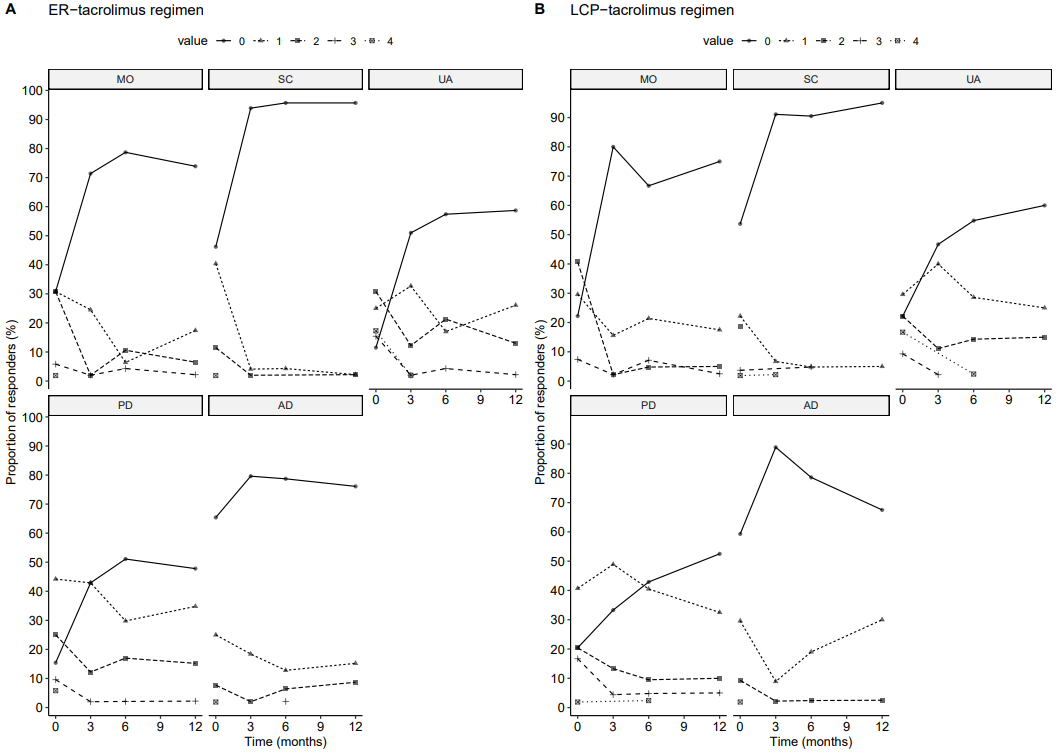


# Supplementary figure 3. SF-36 questionnaire outcomes for every domain


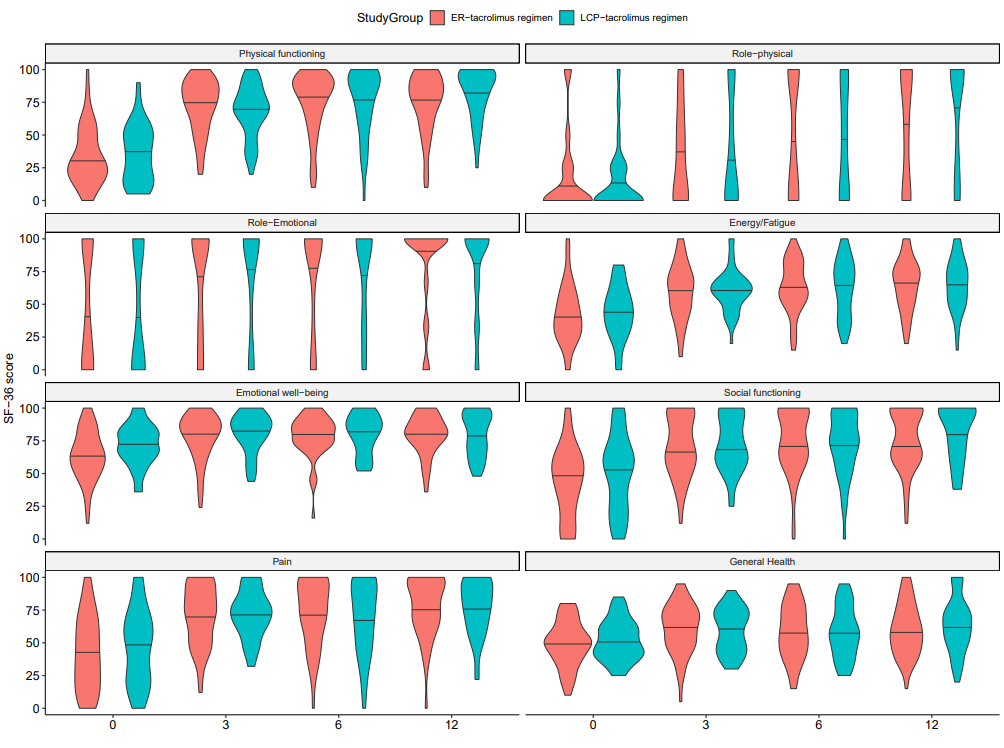


# Supplementary figure 4. Subgroup analysis of the EQ-VAS in relation to tremors


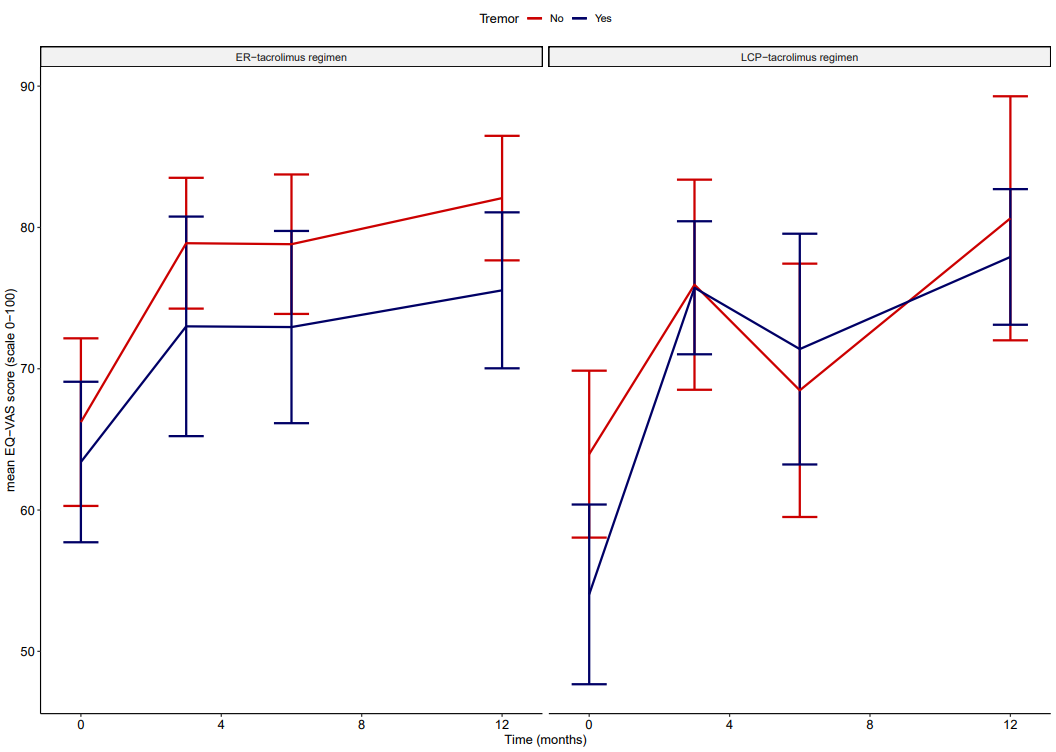


# Supplementary figure 5. Tacrolimus levels versus regimen and the presence of tremors

At month 3 in the ER-tacrolimus regimen 33 patients experienced no tremor and 16 patients experienced tremor, in the LCP-tacrolimus 30 patients experienced no tremor and 15 patients experienced tremor. At month 6 in the ER-tacrolimus regimen 35 patients experienced no tremor and 12 patients experienced tremor, in the LCP-tacrolimus 30 patients experienced no tremor and 12 patients experienced tremor. At month 12 in the ER-tacrolimus regimen 32 patients experienced no tremor and 14 patients experienced tremor, in the LCP-tacrolimus 30 patients experienced no tremor and 10 patients experienced tremor.


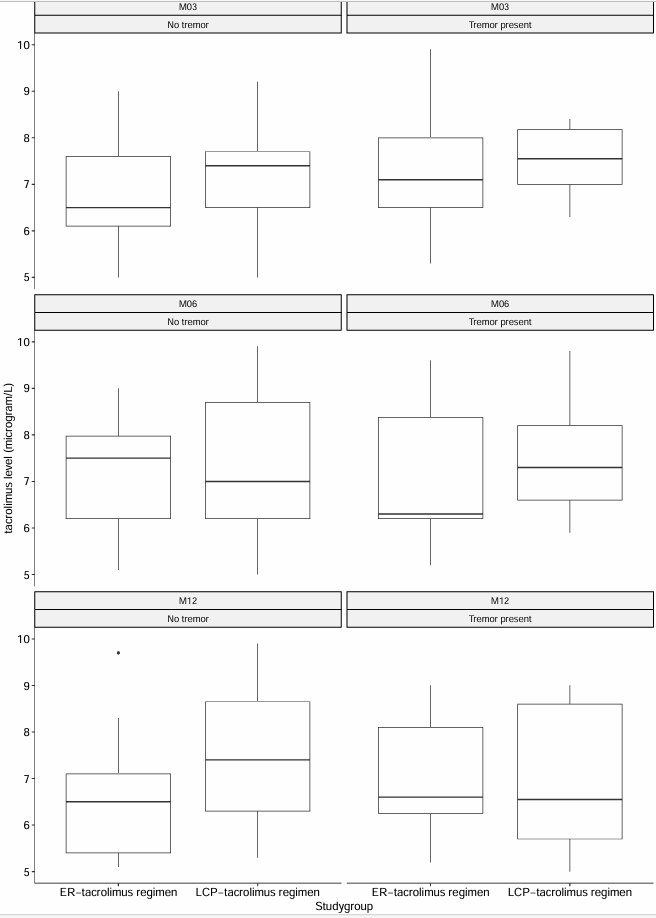

Supplement: Supplementary file 1 [file Table1.DOCX]
